# Climate Change Abatement Strategies Which Way is the Wind Blowing?

**DOI:** 10.1289/ehp.117-a296

**Published:** 2009-07

**Authors:** David C. Holzman

**Affiliations:** **David C. Holzman** writes from Lexington and Wellfleet, Massachusetts, on science, medicine, energy, economics, and cars. He has written for *EHP* since 1996

The mitigation of greenhouse gas emissions, already one hot topic, got even hotter with the 16 June 2009 publication of the White House report *Global Climate Change Impacts in the United States*. “Choices made about emissions reductions now and over the next few decades will have far-reaching consequences for climate-change impacts,” warned the strongly worded report, which emphasized the growing sense that action must be taken soon to avoid catastrophic public health fallout from accelerating climate change—a sense echoed in a proposed ruling by the U.S. Environmental Protection Agency (EPA) seeking authority to regulate greenhouse gases as a potential public health threat.

How best to drive the United States toward mitigation goals is a matter of disagreement among experts and politicians, however. The task is dauntingly complex because so many sources of greenhouse gases exist. The major sources of U.S. emissions are industry at 30%, transportation (including all forms of mass transit and shipping) at 28%, residential and commercial at 17% each, and agriculture at 8%, according to the Pew Center on Global Climate Change. There are other ways to slice the emissions pie. For instance, the electricity industry, which cross-cuts the above sectors, accounts for 30% of U.S. emissions.

Past regulatory efforts aimed at reducing fossil fuel use—which were geared toward problems other than greenhouse gas emissions, such as trade deficits and traffic congestion—illustrate the need for market forces to get the job done. For instance, it was only after gas prices soared past $4 per gallon in summer 2008 that sales of high-mileage cars finally surged, while SUV sales tanked. But gas prices’ tumble back below $2 per gallon has once again dampened demand for economy cars and raised light trucks’ market share back to roughly half, underscoring the mantra of economists, which has gained adherents among the major environmental groups: when it comes to changing human behavior, prices trump rules and regulations.

Thus, almost everyone who is concerned about climate change mitigation favors putting a price on greenhouse gas emissions. The question is, what strategies will yield the most mitigation bang for the investment buck?

## Rules and Regulations: Laced with Loopholes

The history of so-called command-and-control policies—which dictate not only what the regulations are but also how they will be met—illustrates their limitations. The Corporate Average Fuel Economy (CAFE) standards were a logical response to the Arab oil embargo of 1973–1974, which swelled the U.S. trade deficit and saw Americans spending hours in gas lines. CAFE doubled new car fuel mileage to roughly 27.5 mpg by 1985. And that is where fleet-average automobile fuel economy has remained ever since—riddled with loopholes and becalmed by a lack of political will—despite technologic improvements that could probably have raised it by roughly one-third for light trucks and two-thirds for cars, says John DeCicco, a Michigan-based automotive consultant.

Additionally, CAFE spawned the SUV, which was classified for regulatory purposes as a light truck and was thus subject to much lower standards (slightly under 21 mpg) because at the time most were used for commercial purposes and because U.S. automakers lobbied for their exemption. Eventually, light trucks—a category that also includes minivans— grew to more than half the new car market, resulting in a slight decline in fleet-average fuel economy.

It took a convergence of rising worries about the geopolitical and climatologic effects of excessive oil consumption to muster the political will to pass the first significant hike in CAFE standards as part of the Energy Independence and Security Act in December 2007. This hike was supposed to push the fleet average to 35 mpg by 2020, but it included its own loopholes. For instance, it allowed manufacturers to trade credits in the manner of a carbon cap and trade scheme. Car companies could buy unlimited credits from the federal government, meaning they could buy their way out of mileage improvements.

On 19 May 2009 President Obama announced he was superceding that standard with one that will require a fleet average of 35.5 mpg by 2016. Various loopholes have been mitigated, but not eliminated, in Obama’s new standards, says Roland Hwang, vehicles policy director for the Natural Resources Defense Council.

Some jurisdictions promote purchases of high-mileage automobiles by offering benefits to owners of hybrid vehicles such as single-occupant access to high-occupancy vehicle lanes during rush hour. But these policies don’t necessarily encourage drivers to save gas. In metropolitan Washington, DC, for example, the gas-guzzling Chevy Tahoe hybrid SUV (which gets 21 combined city and highway mpg, according to *Consumer Reports*) automatically has access to high-occupancy vehicle lanes whereas the conventionally powered Honda Civic (at 33 combined mpg) and Smart ForTwo coupe (at 36 combined mpg) do not.

Moreover, the cost of abating carbon dioxide (CO_2_) with hybrid technology is a high $100–140 per ton of avoided emissions, according to *Reducing U.S. Greenhouse Gas Emissions: How Much at What Cost?*, a 2007 public–private study produced by groups including Shell Oil, the Natural Resources Defense Council, and the Environmental Defense Fund. By comparison, the report points to numerous measures ranging in cost up to $50 per ton that could be sufficient to cut U.S. carbon emissions projected for 2030 by about one-third, equivalent to a 28% reduction relative to emissions in 2005. In particular, comparable improvement in fuel economy can be achieved in conventionally powered cars—at an overall gain over the lifecycle of the vehicle of $81 per ton of avoided emissions—by using lighter-weight materials, optimal aerodynamics, turbocharging, drivetrain efficiency, and properly inflated tires.

Another example of the limitations of command-and-control legislation is the national Renewable Fuel Standard, adopted under the Energy Policy Act of 2005 and updated in the Energy Independence and Security Act of 2007. This standard called for 9 billion gallons of biomass-based fuel (about 5% of U.S. annual transportation fuel consumption) to be produced beginning in 2008, increasing to 36 billion gallons by 2022. In response, farmers began switching cropland from food to fuel feedstocks, which caused food prices to soar [see “Food vs. Fuel: Diversion of Crops Could Cause More Hunger,” *EHP* 116:A254–A257 (2008)]. Then, in the 29 February 2008 issue of *Science*, Joseph Fargione and colleagues showed that, whereas wild land can store immense amounts of carbon, cultivating new land for crops releases this carbon, creating a “carbon debt” that can last for tens to hundreds of years. In the same issue of *Science*, Timothy Searchinger and colleagues suggested that switching an acre of farmland from food to fuel crops creates demand for new farmland somewhere in the world to make up for that deficit in food production, thereby indirectly contributing to greenhouse gas emissions.

Thus, growing biofuel feedstocks could actually increase greenhouse gas emissions instead of abating them [see “The Carbon Footprint of Biofuels: Can We Shrink It Down to Size in Time?” *EHP* 116:A246–A252 (2008)]. Furthermore, the sustainability of corn ethanol has been questioned repeatedly because the energy required to produce the ethanol—usually derived from fossil fuel—is almost equal to the energy in the ethanol, obviating any presumed emissions or net energy advantage [see “Battle of the Biofuels,” *EHP* 115:A92–A95 (2007)].

In May 2009 the EPA proposed a new standard for renewable fuels that would more rigorously account for the carbon content of fuels (this is called a low-carbon fuel standard). However, EPA Administrator Lisa Jackson said corn ethanol distilleries under construction or already completed would likely be exempt from the new regulations. In addition, the EPA proposed tabulating greenhouse gas emissions over 100 years instead of 30. This would improve corn ethanol’s numbers by allowing more time to pay back the carbon debt incurred when new land is plowed, but 30 years is a far more appropriate basis for analyzing lifecycle carbon impact given the probable urgency of mitigating climate change, says Nathanael Greene, a senior energy policy specialist at the Natural Resources Defense Council. In the 6 May 2009 edition of the *Washington Post*, Frank O’Donnell, head of Clean Air Watch, was quoted as saying, “EPA has left open the option that an exception to good science could be made in the case of a favored special interest.”

The bottom line: it costs roughly 10 times more to achieve a given level of CO_2_ abatement using a low-carbon fuel standard than it does using carbon pricing, according to a study published in the February 2009 issue of *American Economic Journal: Economic Policy* by Stephen Holland of the University of North Carolina at Greensboro and Jonathan Hughes and Christopher Knittel of the University of California, Davis. Additionally, although a low-carbon fuel standard taxes high-carbon fuels, it actually subsidizes low-carbon fuels, thus failing to encourage carpooling, reduced driving, or other carbon avoidance, says Holland, a professor of economics.

In another example of cost-ineffective decision-making in politics, Pennsylvania’s Alternative Energy Portfolio Standard Act of 2004 mandated more than 800 Mw (roughly a nuclear plant’s worth) of solar photovoltaics installations by 2021. But Pennsylvania could obtain the same energy from wind for less than one-quarter the cost, according to “Cap and Trade Is Not Enough: Improving U.S. Climate Policy,” a policy paper from the Department of Engineering and Public Policy, Carnegie Mellon University.

Urban mass transit is another oft-touted solution to greenhouse gas emissions. But several experts, including Andreas Schafer, a lecturer at The Martin Center for Architectural and Urban Studies, University of Cambridge, United Kingdom, believe that outside of densely populated cities, the cost of reducing emissions by luring people out of their cars onto buses or subway systems is far too high relative to other means of mitigation to merit consideration on that basis. “It is very difficult to get people out of their cars and put them into mass transit on a significant scale, whereas improving the fuel efficiency of vehicles is significantly more realistic,” says Schafer.

## To Market, to Market

Whereas a tax simply puts a price on each ton of CO_2_ emitted, under a cap-and-trade system policy makers set a limit on annual carbon emissions, then let the price float, dictated by the market. The government gives or auctions “allowances” (permits to emit a specific quantity of carbon) to CO_2_ emitters, who can buy and sell the allowances among themselves. Thus, a utility that can easily reduce emissions, perhaps through efficiency improvements, can sell its allowances to companies for whom reducing emissions would be more costly than buying the allowances.

Either mechanism—taxation or cap and trade—would best be applied “upstream” at the point of energy production. In other words, instead of having to monitor millions of tailpipes, furnaces, factories, and the like, regulators would oversee “roughly 150 oil refineries, 1,460 coal mines, and 530 natural gas processing plants,” according to *Policy Options for Reducing CO_2_* *Emissions*, a February 2008 report by the Congressional Budget Office.

Between the two market solutions, economists generally prefer a tax because it’s simpler. But it is very hard to change taxation systems, says Gregory P. Nowell, an associate professor of political science at the University at Albany–SUNY. Voters fear they would lose somehow if the taxation system changes, he says, adding that high taxes in Europe are not necessarily due to environmental foresight. “In Europe in the 1930s the coal industry favored punitive taxation on oil to slow that market’s growth,” he says. “But the advantages of oil over coal were so great that the market grew anyhow. Now those taxes account for fifteen to twenty percent of government revenues, and shifting them to other sectors of the economy would be an electoral nightmare.”

Another political advantage for cap and trade is that it has a precedent in the United States, having been used successfully to reduce sulfur dioxide emissions. However, that task, which merely required switching from high- to low-sulfur coal, was far simpler than replacing an entire energy infrastructure, says Laurie Williams, an enforcement attorney with EPA’s region 9, speaking in her personal capacity with ethics clearance from the agency.

The European Union’s greenhouse gas emissions trading scheme (EU ETS), which also lends credibility to U.S. efforts toward cap and trade, nonetheless has often been criticized for “over-allocating” permits—that is, setting the cap higher than current emissions, which can delay measures to reduce emissions. But Denny Ellerman, a senior lecturer in applied economics at the Massachusetts Institute of Technology Sloan School of Management, says this happened during a trial period from 2005 to 2007, and that recently released data for 2008 indicate the scheme, which serves 27 nations, is now reducing emissions.

Still, some critics worry that setting a cap and letting the market determine the price of emissions—rather than setting the price as with a tax—means that when allowance prices fall, so does the incentive for investing in efficient and low-carbon technology, says Michelle Chan, director of the Green Investments Program at the advocacy group Friends of the Earth. A variety of measures can limit that volatility, such as price floors and ceilings, and provisions that allow companies to bank allowances for future years or borrow them, says economist Ian Parry, a senior fellow at the nonprofit Resources for the Future. Nonetheless, economists hold that a ceiling can weaken the cap.

Whichever market mechanism ultimately prevails on Capitol Hill—assuming one does—economists acknowledge the legislation may require complementary measures to offset certain “market failures,” or situations in which the prices of goods do not reflect the true cost of producing those goods. As one example, consumers often fail to consider life-cycle costs of items ranging from light bulbs to houses, or they do so with short several-year horizons in contrast to, say, utility companies, which take a 20- to 30-year view.

These market failures are costly, according to *Reducing U.S. Greenhouse Gas Emissions: How Much at What Cost?* Nearly 40% of abatement could be achieved at negative marginal cost, according to the report. For example, it is cheaper to build efficient buildings, vehicles, and appliances than it is to retrofit or retire them early, yet such options must be pursued quickly because the potential benefit diminishes rapidly as more inefficient buildings and vehicles are produced.

## The American Clean Energy and Security Act of 2009

Market strategies and complementary measures for supporting them are both addressed in the American Clean Energy and Security Act of 2009, sponsored by Representatives Henry Waxman (D–CA) and Edward Markey (D–MA). The bill creates an economy-wide cap-and-trade program at the level of refiners, importers of liquid fuels, and the coal mining industry, augmented by a smorgasbord of complementary measures.

The bill aims to boost the share of low- or zero-carbon primary energy (energy that exists in raw form, such as the coal or uranium used in power plants to generate electricity, or the solar energy that hits a collector, as opposed to the resulting electricity or heat they provide to consumers) to 18% by 2020 and 46% by 2050, according to the EPA’s *Preliminary Analysis of the Waxman–Markey Discussion Draft*. Low- and zero-carbon energy sources include renewable fuels, nuclear power, and fossil fuels with carbon capture and storage measures. The bill also aims to reduce total greenhouse gas emissions by 20% by 2020 and by 83% by 2050, relative to 2005 levels.

If the allowances were auctioned rather than given to CO_2_ emitters, and if most of the revenues from those auctions were given to households, the annual cost of the legislation would be less than $150 per household, according to the EPA analysis. However, the current plan is to give away more than 80% of the allowances, says Williams. In the March 2009 working paper “Who Pays for Climate Policy? New Estimates of the Household Burden and Economic Impact of a U.S. Cap-and-Trade System,” author Andrew Chamberlain of the education group Tax Foundation wrote that a cap-and-trade scheme that begins by giving away allowances would cost the poorest households $528 each versus a net gain of $1,904 to the wealthiest households, thanks to windfall profit dividends from shareholding in the companies receiving free allowances.

The legislation’s current iteration includes a renewable electricity portfolio standard, which would require utilities to obtain 20% of the electricity they produce from renewable sources by 2025 and consider emissions over the entire life-cycle of fuel production and use. There are also provisions for deploying plug-in hybrid electric vehicles (PHEVs) in certain regions. This would include requiring utilities to develop plans for the necessary infrastructure, such as stations for charging and battery swapping. The bill also calls for substantial improvements in building efficiency, lighting, appliances, and investments in public transportation, along with awards for inventions that improve industrial efficiency.

According to the EPA analysis, key uncertainties around the bill include the long-term cost of abatement, the availability and cost of domestic “offset” projects, and the technical, political, and social feasibility of new nuclear power and the large-scale practicality of carbon capture and storage [for more information on this technology see “Carbon Capture and Storage: Blue-Sky Technology or Just Blowing Smoke?” *EHP* 115:A538–A545 (2007)]. The bill also does not address greenhouse gases other than CO_2_ except to make agricultural greenhouse gas emissions a target for offsets.

The major reliance on offsets for roughly one-third of emissions reductions is one of the strongest criticisms of Waxman–Markey. In an offset scenario, polluters can counterbalance their greenhouse gas emissions by paying for carbon-mitigating activities such as planting trees or building a renewable energy installation. The advantage: offsets are cheaper than allowances. In fact, including offsets in the bill is a way to restrain allowance costs.

However, the market for offsets both in the United States and abroad is already unreliable and could get much worse as it rises to a projected $2 trillion annually by 2020, according to Chan. Moreover, the same kind of financial creativity that figured in the recent mortgage market melt-down would likely apply to the offset market. For example, the financial firm Credit Suisse bundled a series of offsets prior to their being verified as legitimate by the United Nations Clean Development Mechanism (which serves parties to the Kyoto Protocol) and then sliced the bundles into packages for sale in a process called “securitization,” says Chan. This same type of activity rendered mortgage-backed securities so far removed from the original loans and the value of the homes they financed that it became impossible to determine the quality of the loans, contributing to the subprime crisis. “[The same] could happen again as carbon securitization deals get bigger and more complex,” says Chan.

In the April 2008 working paper “A Realistic Policy on International Carbon Offsets,” Stanford researchers Michael W. Wara and David G. Victor wrote that corporations seek the cheapest offsets, which also tend to be the ones where mitigation is hardest to measure and verify. Furthermore, they wrote, “much of the current [Clean Development Mechanism] market does not reflect actual reductions in emissions, and that trend is poised to get worse.” [For more information on these schemes, see “Carbon Offsets: Growing Pains in a Growing Market,” *EHP* 117:A62–A68 (2009).]

In devising a renewable electricity portfolio standard, it is important to distinguish among technologies, says Granger Morgan, a professor of engineering and public policy at Carnegie Mellon University. It makes sense, he says, to deploy technologies that are “within striking distance of being cost-competitive,” where growing the market might result in new knowledge that could bring costs down to competitive levels. Conversely, if a technology is far from being cost-competitive and unlikely to achieve it in current form, then investing in research and development toward developing a cost-competitive version makes more sense.

Subsidies make sense for wind power, says Morgan. “Wind is now one of the most cost-effective ways to produce low-carbon electricity.” If utilities had to pay for carbon emissions, electricity would be cheaper from wind than from coal, he says. However, Williams criticizes Waxman–Markey for putting “such a low price on carbon it will not make even wind cost-competitive.”

Subsidies also make sense for 20-mile-range PHEV batteries, both to learn how to improve the infrastructure and to provide incentives for development of better batteries, says Morgan, whereas 60-mile batteries would not be cost-effective at present. Moreover, PHEVs are not appropriate in regions where coal, which supplies half of U.S. electricity, is the major source of electricity, because in these cases, they would not necessarily reduce greenhouse emissions.

Waxman–Markey is likely to pass the House in late 2009 or early 2010, says Juliet Eilperin, a *Washington Post* reporter who covers environmental matters on Capitol Hill. But even with the Senate in Democratic hands, the bill’s passage in that body is by no means assured. Waxman–Markey is somewhat controversial among proponents of a market solution, some of whom favor a carbon tax. It has received praise from some environmentalists (though vehement opposition from others), some economists, and from the EPA analysis—although, says Williams, many agency staff disagree with this analysis. Should Congress fail to act, it would be left to the EPA to regulate greenhouse gases, a process that could take two years, according to an agency spokesperson.

## No Easy Answers

Nowell warns that the tools used for greenhouse gas mitigation must be appropriate for the task. “We are facing what is arguably the greatest environmental calamity in human history with regulatory mechanisms that were designed for other uses, including congestion control and tropospheric pollution control,” he says. “It has a heroic quality, but also resembles trying to wage war against a modern army with pitchforks and baseball bats.”

So what strategy offers the most climate mitigation bang for the investment buck? Among 18 experts questioned on mitigation strategies by the U.S. Governmental Accountability Office for its May 2008 report, *Climate Change: Expert Opinion on the Economics of Policy Options to Address Climate Change*, 7 preferred a tax, and 11 preferred some form of cap and trade. Despite that disagreement, one message came through loud and clear: 16 of the 18 experts urged adoption of some form of carbon pricing as soon as possible.

## Figures and Tables

**Figure:**
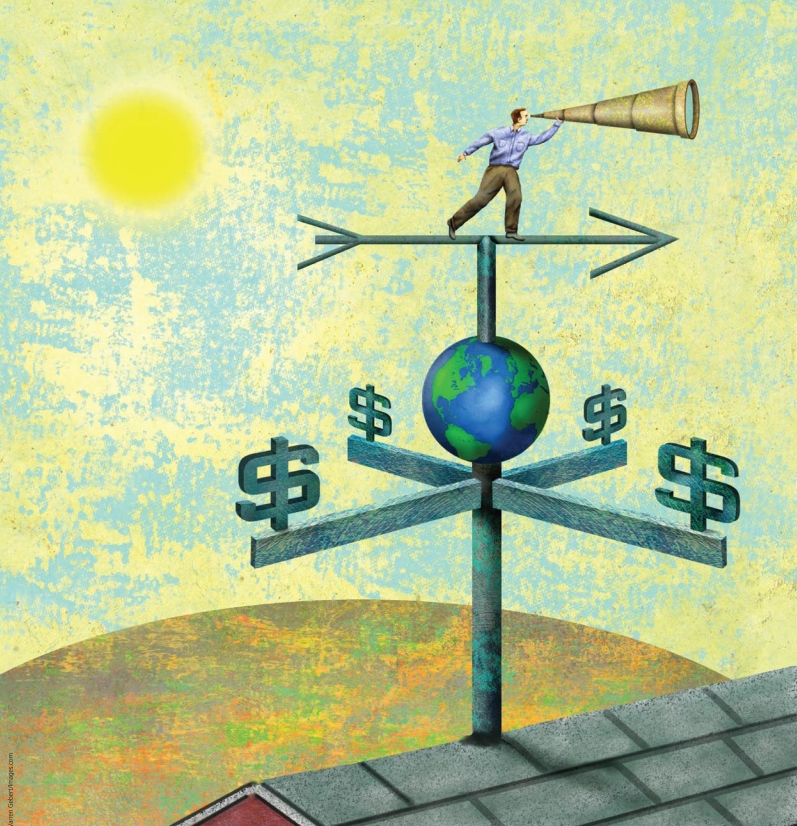


**Figure:**
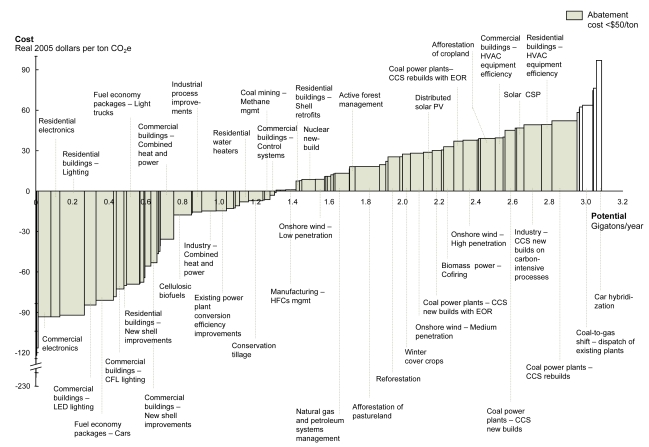
Abatement Options: A Spectrum of Costs In the report *Reducing U.S. Greenhouse Gas Emissions: How Much at What Cost?* a team of economists calculated low-, mid-, and high-range emission reductions by 2030 using a variety of low-cost measures in five different sectors: buildings and appliances, transportation, industry and waste, terrestrial carbon sinks, and power. Under the mid-range or high-range scenarios, emissions could be reduced by 3.0 or 4.5 gigatons, respectively, below 2005 levels. The chart above illustrates a range of abatement options to achieve mid-range reductions by 2030. The tables on the following pages depict mid-range abatement options for specific sectors that could be achieved for less than $50 per ton of avoided emissions while maintaining “comparable levels of consumer utility”—defined as “functionality or usefulness for people, including level of comfort.” Nearly 40% of abatement options could be pursued at a negative marginal cost, meaning that investing in these options would net a positive return over the options’ lifecycle. Charts this page reprinted from Creyts J, et al. 2007. Reducing U.S. Greenhouse Gas Emissions: How Much at What Cost? New York, NY: McKinsey & Company; p. 16 (right); p. 20 (above).

**Figure:**
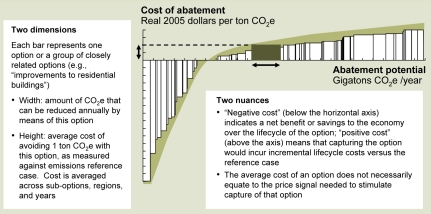
How to Read an Abatement Curve Avoided emissions are shown above and in the tables that follow as tons of CO_2_e, or carbon dioxide equivalents, the standard unit for reporting CO_2_ emissions. Abbreviations: CCS = carbon capture and storage CFL = compact fluorescent lamp CSP = concentrated solar power EOR = enhanced oil recovery HFC = hydrofluorocarbon HVAC = heating, ventilating, and air conditioning LED = light-emitting diode PV = photovoltaic

**Figure:**
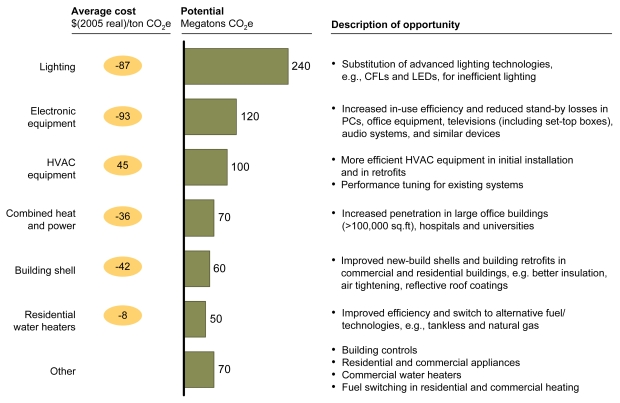
Abatement Options: Buildings and Appliances Building shell refers to the outer construction of a building (walls, insulation, windows, foundation, etc.). Combined heat and power refers to use of a single system to produce both electricity and heat. Abbreviations: CFL = compact fluorescent lamp; HVAC = heating, ventilating, and air conditioning; LED = light-emitting diode; PC = personal computer. Reprinted from Creyts J, et al. 2007. Reducing U.S. Greenhouse Gas Emissions: How Much at What Cost? New York, NY: McKinsey & Company; p. 36.

**Figure:**
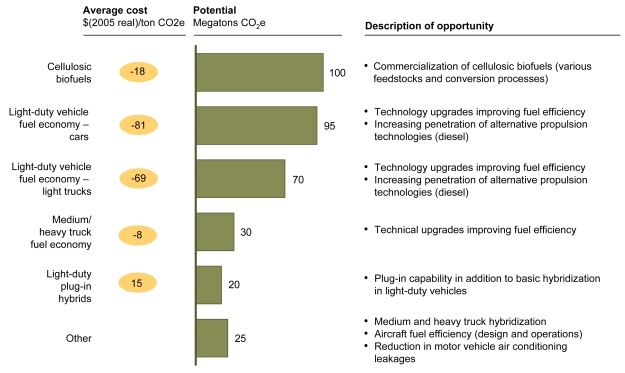
Abatement Options: Power Carbon capture and storage refers to capturing carbon emissions and sequestering them to prevent their contributing to climate change. Conversion efficiency refers to the amount of input required to produce usable energy. Up-rating refers to increasing a power plant’s generating capacity. Abbreviations: CCS = carbon capture and storage; PV = photovoltaic. Reprinted from Creyts J, et al. 2007. Reducing U.S. Greenhouse Gas Emissions: How Much at What Cost? New York, NY: McKinsey & Company; p. 59.

**Figure:**
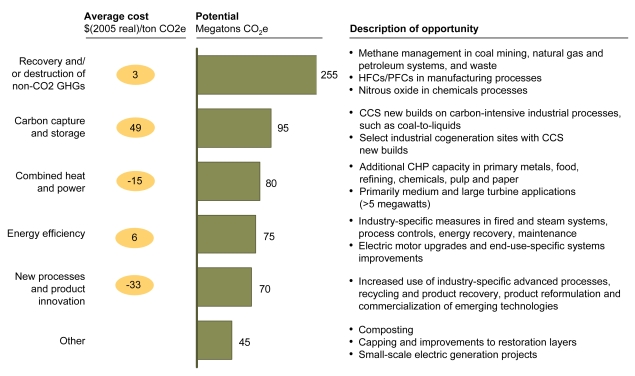
Abatement Options: Transportation Cellulosic biofuels refers to fuels derived from the cellulose that makes up the woody part of plants (in contrast, fuels such as corn ethanol are derived from starches). Reprinted from Creyts J, et al. 2007. Reducing U.S. Greenhouse Gas Emissions: How Much at What Cost? New York, NY: McKinsey & Company; p. 43.

**Figure:**
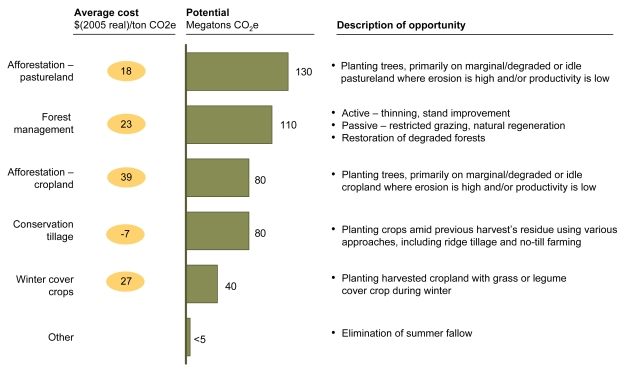
Abatement Options: Industry and Waste Combined heat and power refers to use of a single system to produce both electricity and heat. Carbon capture and storage refers to capturing carbon emissions and sequestering them to prevent their contributing to climate change. Abbreviations: CCS = carbon capture and storage; CHP = combined heat and power; GHG = greenhouse gas; HFC = hydrofluorocarbon; PFC = perfluorocarbon. Reprinted from Creyts J, et al. 2007. Reducing U.S. Greenhouse Gas Emissions: How Much at What Cost? New York, NY: McKinsey & Company; p. 50.

**Figure:**
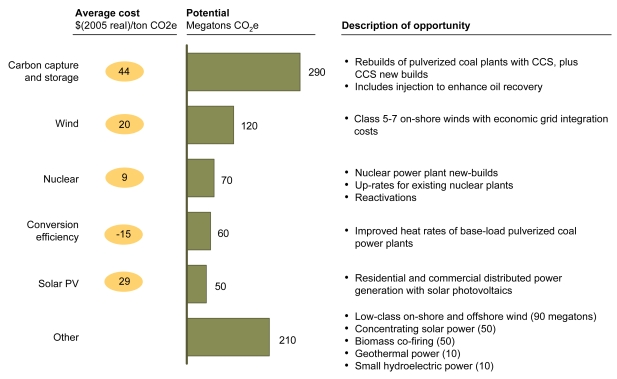
Abatement Options: Terrestrial Carbon Sinks Afforestation refers to planting forests on lands that have not historically been used for this purpose. Tillage refers to methods of tilling or turning the soil. Stand improvement refers to methods for optimizing the growth of desired trees (for instance, by removing less desirable trees that compete for sunlight). Summer fallow refers to letting cropland lie dormant over the summer to recoup soil moisture for winter crops. Reprinted from Creyts J, et al. 2007. Reducing U.S. Greenhouse Gas Emissions: How Much at What Cost? New York, NY: McKinsey & Company; p. 55.

